# IRA-YOLO: Inter-Class Relation Attention for PPE Detection in Industrial Scenes

**DOI:** 10.3390/s26185882

**Published:** 2026-09-17

**Authors:** Ran Zhao, Haoran Duan, Xiaodong Liu, Nan Xu, Junlin Du, Pei Zhou

**Affiliations:** 1 Shandong Hi-Speed Construction Management Group Co., Ltd., Jinan 250098, China; zr9999@wo.cn (R.Z.); qq602596807@gmail.com (H.D.); xd.liu-sdhs@foxmail.com (X.L.); 2 Anshi Technology Co., Ltd., Chengdu 610000, China; xunan@anshi-tech.com.cn; 3 College of Computer Science, Sichuan University, Chengdu 610065, China; dujunlin@stu.scu.edu.cn

**Keywords:** personal protective equipment detection, object detection, attention mechanism, inter-class relation modeling

## Abstract

Personal protective equipment (PPE) detection in industrial scenes is a joint classification-and-localization problem complicated by partial occlusion, scale variation, and background clutter. PPE categories also follow structured physical relations; for example, helmets are normally associated with heads and gloves with hands. Existing attention modules mainly reweight appearance features and seldom encode these directional inter-class dependencies. We therefore propose IRA-YOLO, a YOLO-based object detector equipped with an Inter-Class Relation Attention Module (IRAM). The IRAM projects each multi-scale feature map into class-specific subspaces, learns asymmetric pairwise relations among category attention maps, and returns the relational prior to the detection feature through residual fusion. On SH17, IRA-YOLO improves mAP@50:95 from 0.396 to 0.411 over YOLOv11s while adding 0.42 M parameters and 0.99 GFLOPs, and it maintains 139.83 FPS in the single-image inference test. Cross-dataset evaluation on CHVG further supports the transferability of the proposed feature refinement. These results indicate that explicit inter-class relation modeling provides complementary semantic context for multi-class PPE detection.

## 1. Introduction

Industrial and construction environments expose workers to substantial safety risks, and correct use of personal protective equipment (PPE) is an important means of reducing injuries and associated economic losses. Manual inspection is labor-intensive, subjective, and difficult to scale. As a result, vision-based non-contact monitoring has been widely explored for workplace safety management [1,2].

Real-time one-stage detectors provide a practical foundation for such monitoring systems. In particular, the YOLO family has evolved rapidly from the widely adopted YOLOv5 [3] and YOLOv8 [4] implementations to more recent architectures such as YOLOv9 [5], which introduces programmable gradient information, and the end-to-end YOLOv10 detector [6]. These advances have progressively improved the balance between detection accuracy and computational efficiency, making YOLO-based models attractive for continuous PPE monitoring in industrial environments.

Despite these advances, reliable PPE detection in real industrial scenes remains challenging because of scale variation, illumination changes, background clutter, occlusion, and small target size. Moreover, PPE categories do not occur independently but exhibit characteristic semantic and spatial dependencies arising from their intended use on the human body. For example, a helmet is typically associated with the head region, gloves with the hands, and a safety vest with the torso. Such dependencies provide contextual cues for distinguishing plausible PPE configurations from visually similar but semantically inconsistent cases, such as a helmet located near the head versus one located near the hand. They are particularly useful when local appearance cues are unreliable because of occlusion, small object size, or visual ambiguity. Furthermore, these dependencies can be directional: evidence from one category may contribute differently to another category depending on their semantic and spatial context. This observation motivates the explicit modeling of inter-class relations for PPE detection.

To enhance feature representation, a wide range of attention mechanisms have been introduced in recent years. Channel attention methods, such as SENet [7], and joint channel–spatial attention methods, such as CBAM [8], improve the discriminability of visual features by reweighting local responses or global statistics. However, such mechanisms primarily emphasize informative features based on appearance responses and do not explicitly exploit the semantic dependencies among PPE categories described above. Relation-aware approaches, including graph convolutional networks [9] and Relation Networks [10], can model object interactions more explicitly, but they often introduce non-negligible computational overhead and may be less compatible with real-time industrial deployment.

To address these limitations, we propose IRA-YOLO, an inter-class relation attention framework built upon YOLOv11 [11]. Rather than treating category responses independently, IRA-YOLO exploits learned inter-class dependencies to complement appearance-based evidence and refine category-specific feature responses. The proposed Inter-Class Relation Attention Module (IRAM) consists of three stages: (1) class-specific attention map generation via subspace projection, (2) pairwise inter-class relation modeling through grouped convolutions, and (3) relation-guided feature refinement. The main contributions of this work are summarized as follows:

1. We propose the IRAM, a proposal-free module that models asymmetric pairwise semantic dependencies among PPE categories directly in dense feature maps and fuses the resulting relational information into appearance features through a residual connection. Unlike proposal-level graph reasoning, the IRAM operates before the detection head and does not require candidate boxes.
2. We design a class-specific multi-subspace projection and grouped-convolution relation pathway that separates category evidence before learning directional interactions, thereby limiting the additional parameter and computational costs.
3. We evaluate the method on SH17 [12], compare it with representative detectors and attention modules, assess its transfer performance on CHVG, and report an additional real-time inference benchmark. The remaining condition-stratified analyses requested during review are explicitly identified in Section 4.6.

The remainder of this paper is organized as follows: Section 2 reviews related work on PPE detection, attention mechanisms, and relation modeling. Section 3 introduces the proposed IRA-YOLO framework and the IRAM design. Section 4 reports the experimental settings and results. Section 5 concludes the paper.

## 2. Related Work

### 2.1. Personal Protective Equipment Detection

PPE detection has attracted increasing attention because of its direct relevance to industrial safety management. Early methods relied on handcrafted image processing techniques, such as color thresholding and edge detection [1]. However, such approaches are highly sensitive to illumination variation, viewpoint changes, and background clutter.

With the development of deep learning, convolutional neural network-based detectors have become a common solution. Two-stage detectors such as Faster R-CNN [13] generally achieve high accuracy, but their inference cost limits real-time deployment. By contrast, single-stage detectors, especially the YOLO family [14,15,16], offer a balance between accuracy and efficiency for online monitoring. Beyond the mainstream YOLO lineage, YOLOX introduced a decoupled head and anchor-free design [17], while Mamba-YOLO incorporated selective state-space modeling to capture long-range dependencies efficiently [18]. Recent studies have applied YOLO variants to PPE detection on datasets such as SWHD [12], SHD [2], and SH17 [12], indicating that this detector family is applicable to PPE-related tasks.

End-to-end Transformer detectors provide another important direction. RT-DETR eliminates the need for NMS and demonstrates that DETR-based models can achieve competitive real-time performance relative to YOLO detectors [19]. Nevertheless, its comparatively high computational cost can restrict deployment on resource-constrained industrial platforms, motivating continued research into lightweight one-stage architectures.

Nevertheless, most existing PPE detectors mainly improve backbone design, neck structures, or data augmentation strategies, while paying limited attention to the semantic dependencies among PPE categories. This omission becomes more problematic in complex industrial scenes, where appearance cues alone may be insufficient for reliable detection.

### 2.2. Attention Mechanisms in Object Detection

Attention mechanisms allow neural networks to emphasize informative responses while suppressing irrelevant features. SENet [7] introduced channel-wise recalibration through global pooling and fully connected layers, and CBAM [8] further combined channel and spatial attention. Subsequent studies proposed lightweight alternatives, including ECA [20] and coordinate attention [21], to reduce the computational burden. For object detection, BAM [22] introduced bottleneck attention modules, SimAM [23] presented a parameter-free attention mechanism motivated by neuroscience, and SA-Net [24] combined multiple attention strategies within a lightweight design. Despite their utility, these methods mainly reweight features within channels or spatial locations and do not explicitly model dependencies among object categories, which is particularly relevant to PPE detection, where semantic co-occurrence frequently provides useful contextual information.

### 2.3. Relation Modeling in Computer Vision

Modeling relationships among objects has been studied in several computer vision tasks. Relation Networks [10] introduced relation-aware representations for few-shot learning. Graph convolutional networks (GCNs) [9] provide a natural way to propagate information over predefined or learned nodes and have therefore been used for scene graph generation [25], visual question answering [26], and object detection [27]. In object detection, graph reasoning usually treats object proposals, region features, or category embeddings as graph nodes, and then refines these nodes through an adjacency matrix before classification or localization. This design explicitly captures object–context dependencies, but it also introduces a separate graph construction stage and can depend on the quality of proposals or detected regions. Transformer-based architectures [28] further advanced relation modeling through self-attention, but their quadratic computational complexity can restrict real-time deployment, while recent state-space models such as Mamba [29] offer another route for long-range dependency modeling.

Our work differs from these relation-modeling approaches in two important respects: First, GCN-based and Relation Network-based methods typically operate at the instance, proposal, or node level, whereas the IRAM operates directly in the dense feature-map space before the detection head. No candidate boxes are required, and the relational prior is injected as an early, detection-agnostic refinement step. Second, the IRAM explicitly models ordered pairwise influence ($R_{i,j}\neq R_{j,i}$ in general; see Section 3) together with residual feature-space fusion. This is important for PPE detection because the presence of a head can support helmet detection more strongly than the reverse direction. To address the reviewer’s concern about relation-modeling comparisons, we also added a GCN-style relation baseline in the revision experiments. This baseline is inserted at the same three feature scales as the IRAM, uses class attention maps as graph nodes, applies a learnable class-to-class adjacency matrix, and is trained under the same SH17 split and optimization settings. Table 1 summarizes the qualitative comparison.

In PPE monitoring, the relation prior has a physical interpretation. A visible head region constrains the plausible location and appearance of a helmet, hands constrain gloves, and a torso region constrains a safety vest. These relations are directional because evidence for a body part can support an equipment category more strongly than the reverse. The IRAM uses this prior as contextual evidence and does not replace localization or box regression.

## 3. Method

### 3.1. IRA-YOLO Overview

**Figure 1:**
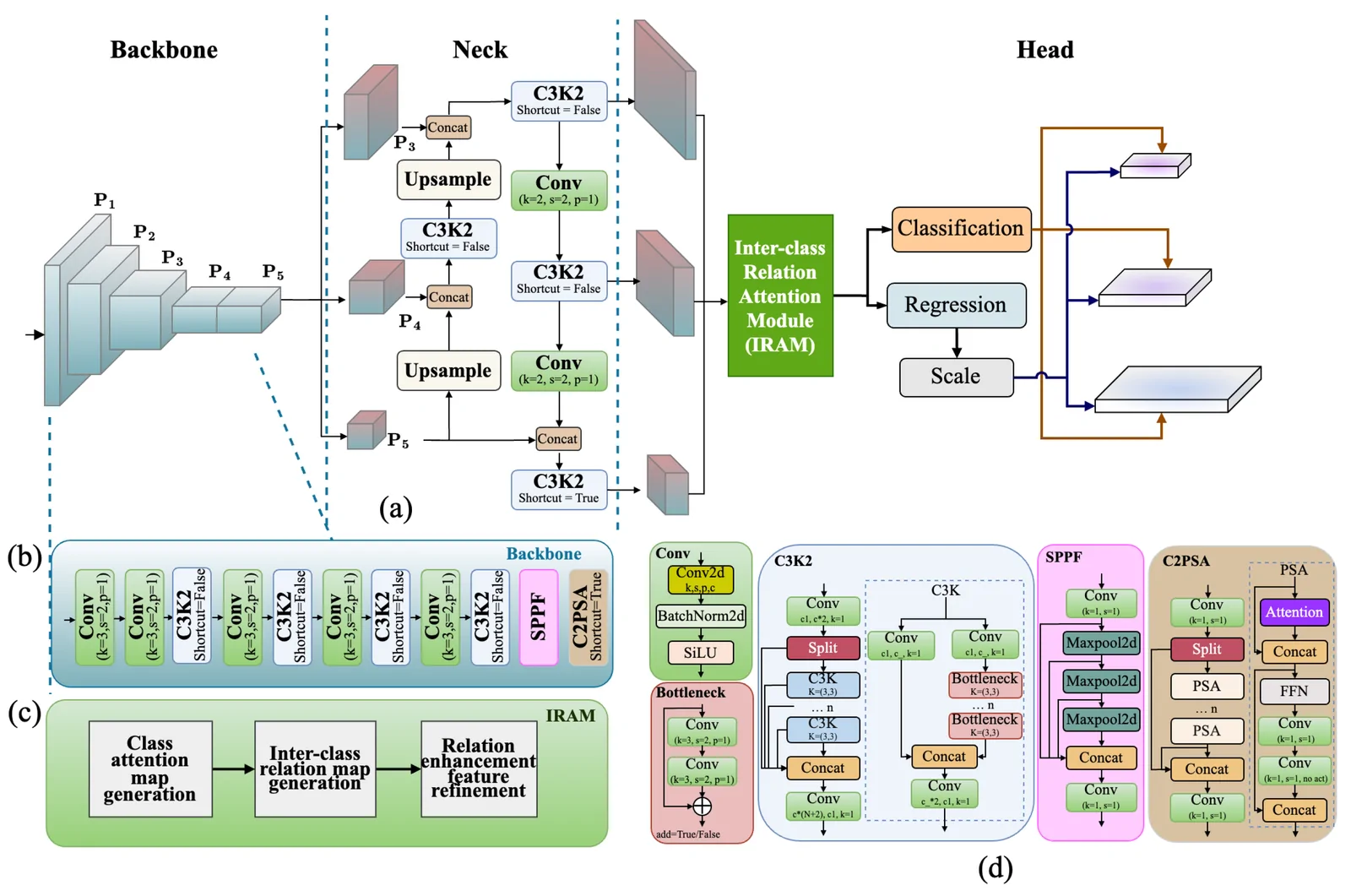
Overview of the proposed IRA-YOLO architecture. (**a**) Overall network architecture, consisting of the backbone, neck, Inter-Class Relation Attention Module (IRAM), and detection head. (**b**) Detailed configuration of the backbone. (**c**) Overall workflow of IRAM, including class attention map generation, inter-class relation map generation, and relation-enhanced feature refinement. (**d**) Detailed structures of the basic modules used in the backbone shown in (**b**), including Conv, Bottleneck, C3K2, SPPF, and C2PSA. The arrows with different colors indicate different feature-flow paths or module connections in the network. The blue dashed lines denote auxiliary information flow or relation-guided feature refinement, and the blue dashed-line boxes highlight the main functional modules or processing stages. The ellipses indicate repeated or omitted intermediate structures for visual clarity and do not affect the scientific understanding of the proposed architecture.

The proposed IRA-YOLO framework is built upon YOLOv11 [11], which serves as the baseline architecture in this study. As illustrated in Figure 1, the IRAM is inserted before each of the three detection heads corresponding to different feature scales.

IRA-YOLO remains an object detector rather than a separate classification or regression model. Each YOLO head predicts class confidence and bounding-box coordinates at one feature scale. The IRAM only refines the shared input feature of that head, so its relational signal can affect both category discrimination and localization through the standard detection losses.

Given an input image, the backbone extracts multi-scale feature maps. At each detection scale, the IRAM processes the feature tensor through three stages: (1) class attention map generation, (2) inter-class relation map generation, and (3) relation-enhanced feature refinement. The refined features are subsequently fed to the detection head for final prediction.

Let $N_{c}$ denote the number of object categories in the detection task. For an input feature map $F\in R^{C_{\mathrm{in}}\times H\times W}$, where $C_{\mathrm{in}}$, *H*, and *W* denote the channel number, height, and width, respectively, the IRAM outputs a refined feature map $F_{\mathrm{out}}\in R^{C_{\mathrm{in}}\times H\times W}$ with identical spatial resolution and channel dimensionality. Table 2 summarizes the dimensionality of the main tensors used throughout this section.

Here, R denotes the set of real numbers and Z denotes the set of integers. We use the single symbol CN for the number of foreground categories throughout the revised formulation; the LaTeX macro $N_{c}$ renders this quantity consistently.

### 3.2. Class Attention Map Generation

**Figure 2:**
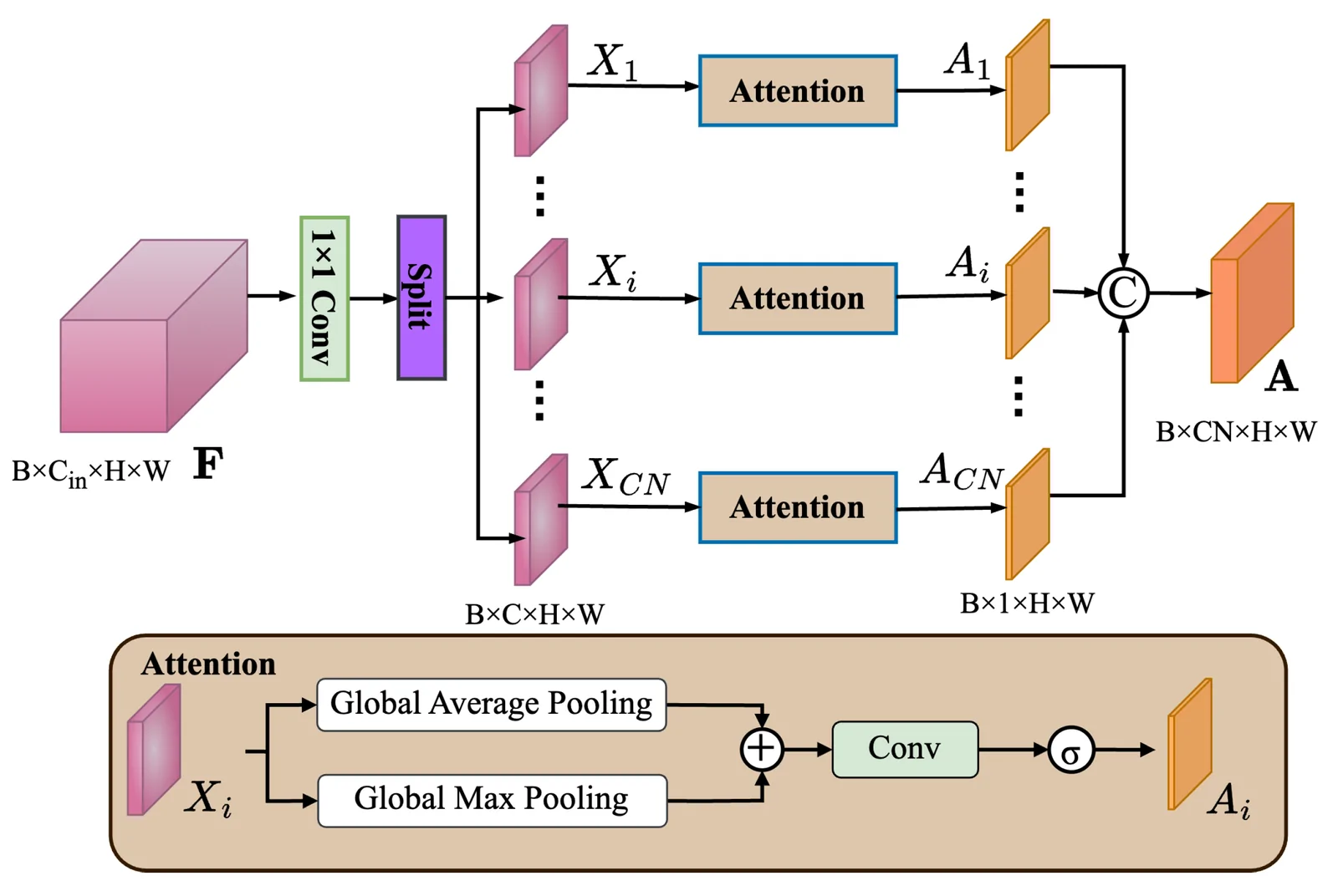
Generation of class attention maps. The input feature tensor is projected into $N_{c}$ class-specific subspaces, and each subspace is converted into a spatial attention map that estimates the distribution of one PPE category.

The first stage of the IRAM decomposes the shared multi-class feature map into category-specific spatial distributions. Inspired by multi-head attention in Transformers [28], we adopt a subspace projection strategy. As shown in Figure 2, we first project the input feature map into a higher-dimensional space that can accommodate independent representations for all categories:

(1) $$F^{\prime }={\mathrm{Conv}}_{1\times 1}\left(F\right)\in R^{d\cdot N_{c}\times H\times W}$$

where $d=2^{n}$ (n∈Z≥0) denotes the channel dimension of each class-specific subspace. In practice, $d\cdot N_{c}$ is selected to be close to $C_{\mathrm{in}}$ in order to balance representation capacity and computational cost. The projected feature map $F^{\prime }$ is then evenly split along the channel dimension into $N_{c}$ independent sub-feature maps:

The three detection scales use separate projections because their input channel dimensions differ. For each scale, *d* is selected as a power of two such that dCN remains close to $C_{\mathrm{in}}$. For the 17-class SH17 model, the P3, P4, and P5 feature maps use $\left(C_{\mathrm{in}},d\right)=\left(128,8\right)$, (256,16), and (512,32), respectively. Their projected channel dimensions $dN_{c}$ are therefore 136, 272, and 544.

(2) $$F^{\prime }=\left[X_{1},X_{2},\ldots ,X_{N_{c}}\right],\;X_{i}\in R^{d\times H\times W}$$

where each sub-feature map $X_{i}$ corresponds to the latent representation associated with the *i*-th category.

For each category-specific feature subspace $X_{i}$, spatial attention is applied to emphasize informative regions:

(3) $$A_{i}=\mathrm{Sigmoid}\left({\mathrm{Conv}}_{3\times 3}\left(\mathrm{Concat}\left[\mathrm{GAP}\left(X_{i}\right),\mathrm{GMP}\left(X_{i}\right)\right]\right)\right)\in R^{1\times H\times W}.$$

Here, GAP and GMP denote channel-wise global average pooling and global max pooling, respectively. Given $X_{i}\in R^{d\times H\times W}$, both operations aggregate the channel responses while preserving the spatial resolution, producing two single-channel spatial descriptors. These descriptors are concatenated along the channel dimension and processed by a 3×3 convolution to integrate local spatial information. The resulting response is normalized by the Sigmoid function to obtain the category-specific attention map $A_{i}\in R^{1\times H\times W}$.

(4) $$A=\left[A_{1},A_{2},\ldots ,A_{N_{c}}\right]\in R^{N_{c}\times H\times W}$$

This tensor encodes the initial spatial distribution estimates for all categories and serves as the input to the subsequent relation modeling stage.

### 3.3. Inter-Class Relation Map Generation

**Figure 3:**
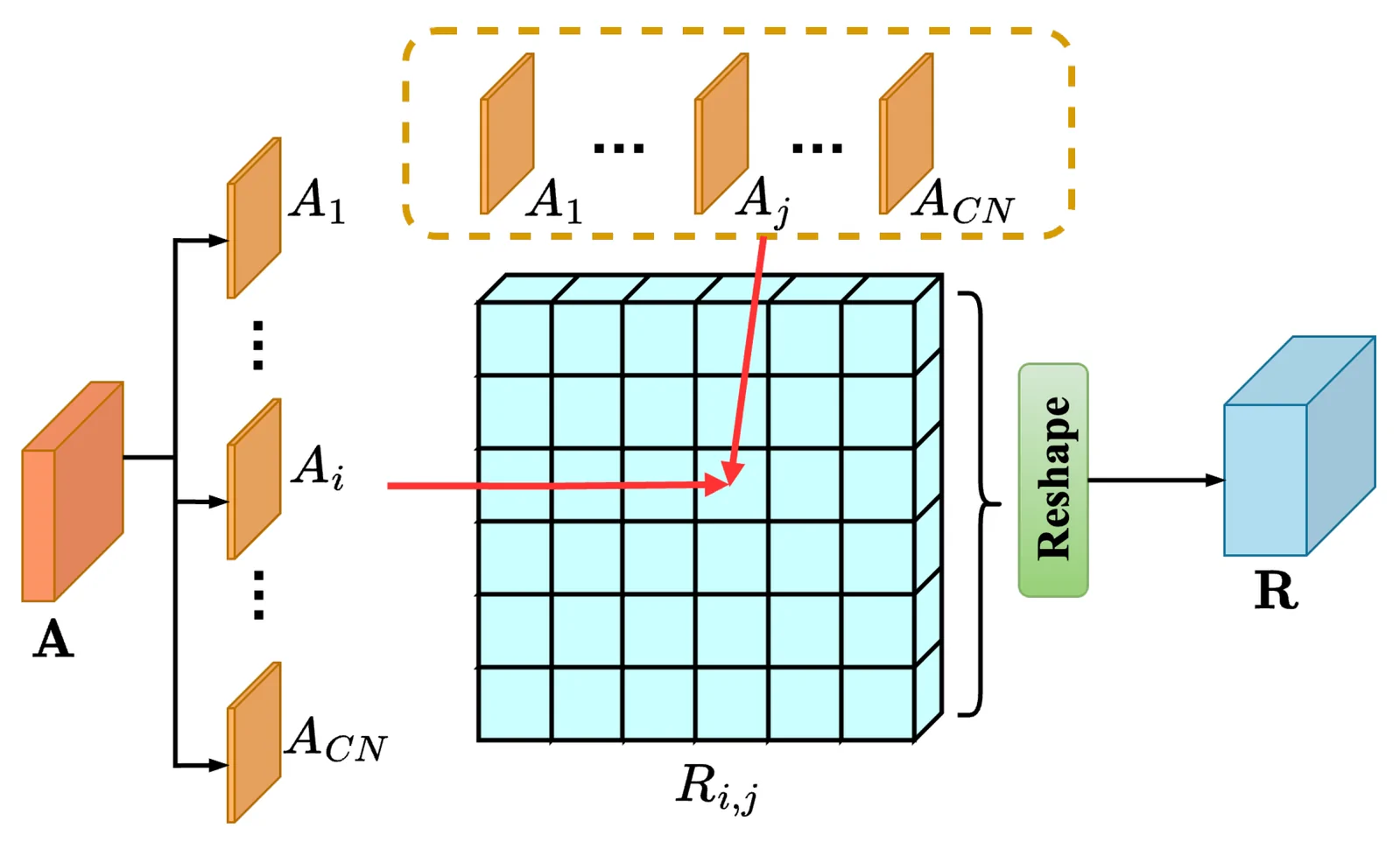
Generation of inter-class relation maps. Pairwise category interactions are modeled by grouped convolutions, yielding $N_{c}\times N_{c}$ asymmetric relation maps that encode the influence of one category on another. The red arrow indicates the directed interaction or influence from one category-specific feature/attention map to another during relation-map generation.

The second stage learns pairwise dependencies among all category pairs and captures asymmetric semantic interactions. Figure 3 illustrates the generation process of the inter-class relation maps, where directed pairwise interactions are constructed between category-specific attention maps. For each category *i*, we construct pairwise interaction features with all categories, including itself, by channel-wise interleaved concatenation:

(5) $$F_{i}={\mathrm{Concat}}_{j=1}^{N_{c}}\left[A_{i},A_{j}\right]\in R^{2N_{c}\times H\times W}.$$

For each source category *i*, its attention map $A_{i}$ is paired with every target attention map $A_{j}$, $j=1,\ldots ,N_{c}$, by channel-wise concatenation. The resulting $N_{c}$ ordered pairs are further concatenated along the channel dimension to form $F_{i}$. This construction encodes all source–target relations (i,j) associated with category *i*, including the self-relation (i,i).

This formulation preserves spatial correspondence between source category *i* and each target category *j*. To enlarge the effective receptive field without incurring the cost of a large kernel, we use three stacked 3×3 convolution layers, which approximate the receptive field of a 7×7 kernel with fewer parameters. Grouped convolutions are further adopted to preserve the independence of different category-pair interactions:

(6) $$R_{i,j}=\mathrm{Conv}\left(\mathrm{Conv}\left(\mathrm{Conv}\left(\mathrm{Concat}[A_{i},A_{j}]\right)\right)\right)\in R^{1\times H\times W}$$

where $R_{i,j}$ represents the spatial influence weight from category *i* to category *j*. For each category *i*, the complete set of outgoing relations is $R_{i}=\left[R_{i,1},R_{i,2},\ldots ,R_{i,N_{c}}\right]\in R^{N_{c}\times H\times W}$, and stacking relations for all categories yields the full inter-class relation tensor:

This construction plays the role of a category-wise multi-head mechanism. Each source category *i* defines one relation head, and grouped operations process all CN source–target pairs in parallel without mixing pairs prematurely. Because the ordered inputs $\left[A_{i},A_{j}\right]$ and $\left[A_{j},A_{i}\right]$ differ, the learned relations need not be symmetric.

(7) $$R=\left[R_{1},R_{2},\ldots ,R_{N_{c}}\right]\in R^{N_{c}\cdot N_{c}\times H\times W}$$

This tensor encodes all $N_{c}\times N_{c}$ asymmetric pairwise relationships and provides semantic priors for feature enhancement.

### 3.4. Relation-Enhanced Feature Refinement

**Figure 4:**
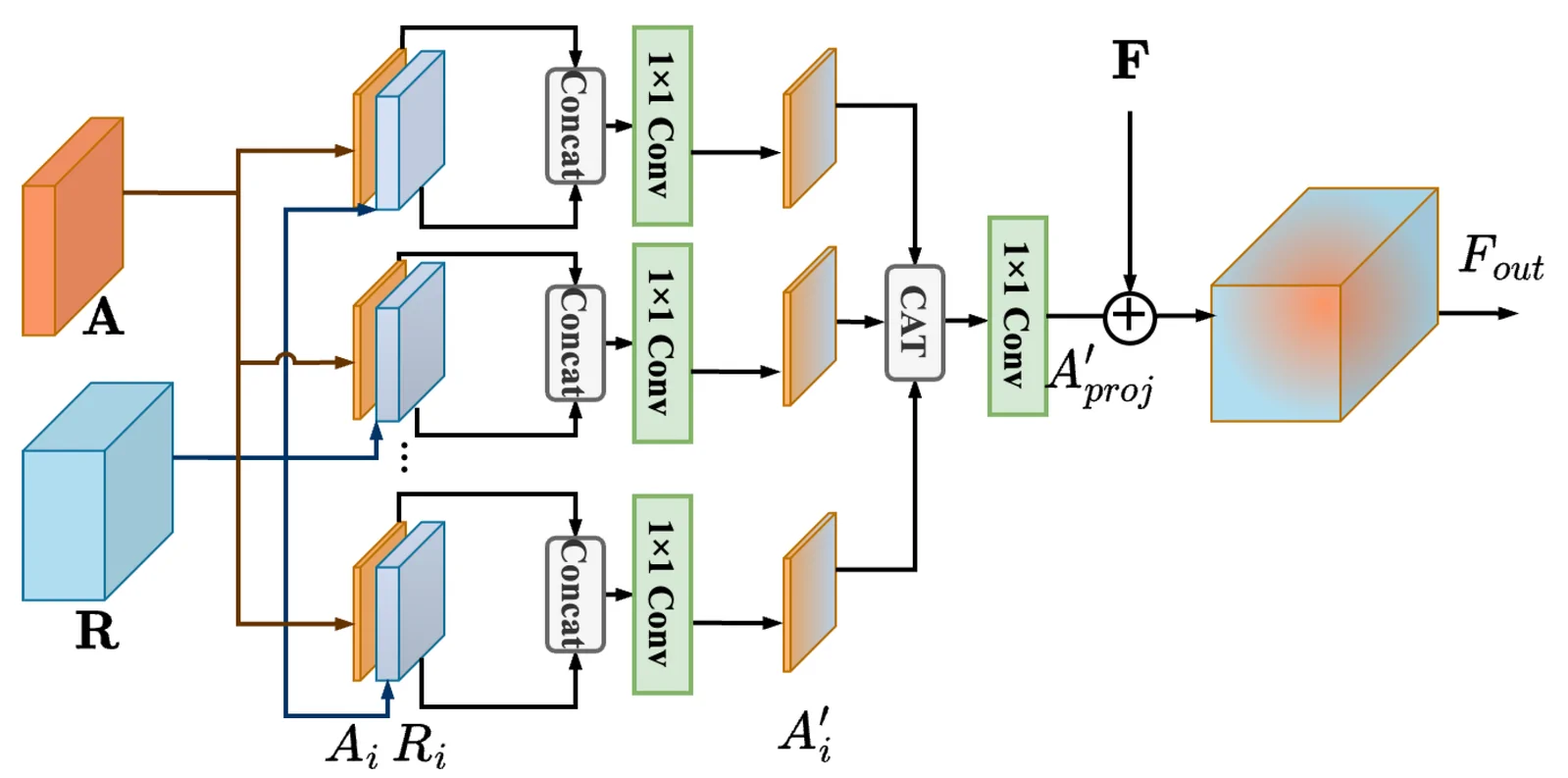
Relation-enhanced feature refinement. For each category *i*, the initial attention map $A_{i}$ is concatenated with the corresponding inter-class relational representation $R_{i}$ and refined by a 1×1 convolution to obtain $A_{i}^{\prime }$. The category-wise refined maps are concatenated and projected back to the original feature space to produce $A_{\mathrm{proj}}^{\prime }$, which is then fused with the input feature *F* through element-wise residual addition, yielding the relation-enhanced output feature $F_{\mathrm{out}}$.

The final stage uses the learned inter-class relations to refine the initial class attention maps and generate the output features. As shown in Figure 4, this stage fuses the initial attention map $A_{i}$ with the relational tensor $R_{i}$ and projects the refined maps back to the original feature space. For category *i*, the initial attention map $A_{i}$ may be weak or missing in challenging scenarios such as occlusion, motion blur, or small objects. We correct this by fusing the relational tensor $R_{i}$ with the original attention:

(8) $$A_{i}^{\prime }={\mathrm{Conv}}_{1\times 1}\left(\mathrm{Concat}[A_{i},R_{i}]\right)\in R^{1\times H\times W}$$

This fusion allows the network to complement weak visual evidence with cues from semantically related categories. The corrected attention maps $A^{\prime }=\left[A_{1}^{\prime },\ldots ,A_{N_{c}}^{\prime }\right]$ reside in semantic space ($N_{c}$ channels) and are projected back to the original feature embedding space via

(9) $$A_{\mathrm{proj}}^{\prime }={\mathrm{Conv}}_{1\times 1}\left(A^{\prime }\right)\in R^{C_{\mathrm{in}}\times H\times W}$$

To ensure training stability and preserve the original information, we employ a residual connection:

(10) $$F_{\mathrm{out}}=F+A_{\mathrm{proj}}^{\prime }\in R^{C_{\mathrm{in}}\times H\times W}$$

Here, *F* and $A_{\mathrm{proj}}^{\prime }$ are combined by element-wise addition, as they share the same spatial and channel dimensions. This residual connection retains the original feature representation while injecting the relation-enhanced attention response. The resulting feature $F_{\mathrm{out}}$, containing both visual and inter-class relational information, is then forwarded to the detection head.

## 4. Experiments

### 4.1. Dataset and Implementation Details

Experiments were conducted on the SH17 dataset [12] ($N_{c}=17$ categories), a large-scale open-source PPE benchmark containing 8099 real-world images collected from diverse industrial environments and 75,994 annotated instances across 17 categories (person, ears, ear muffs, face, face mask, mask, feet, tools, glasses, gloves, helmet, hands, head, medical overalls, shoes, overalls, and safety vest). Compared with other PPE datasets (Table 3), SH17 includes broader category coverage and a larger sample size, making it appropriate for evaluating multi-category PPE detectors.

We adopted an 8:2 split, resulting in 6479 training images and 1620 test images. YOLOv11s [11] was used as the baseline model. The IRAM was inserted before each of the three detection heads. All input images were resized to 640 × 640. The models were trained for 400 epochs with a batch size of 32 using SGD and a OneCycleLR schedule, with an initial learning rate of 0.01 and a final learning rate of 0.0001. Early stopping with a patience of 100 epochs was adopted to reduce overfitting. Standard data augmentation, including random cropping, rotation, and color jittering, was applied during training. All experiments were conducted with PyTorch 2.1 on an NVIDIA RTX 4090 GPU. For the real-time inference evaluation reported in Table 4, FPS was measured separately on an NVIDIA RTX 3090 GPU with a batch size of 1. All compared models were evaluated under the same inference setting to ensure a consistent comparison of computational efficiency.

### 4.2. Comparison with State of the Art

The SH17 split was image-disjoint and used an 8:2 train/test ratio, with 6479 training images and 1620 test images. No cross-fold validation was used; the test set was reserved for final evaluation. The study was an object-detection study: the detection heads jointly optimized classification confidence, localization, and bounding-box regression, while the IRAM supplied contextual feature refinement.

For deployment reporting, FPS and latency were measured after 50 warm-up iterations with a fixed 640 × 640 input, batch size one, FP32 precision, CUDA synchronized timing, and identical pre-processing and post-processing settings on the same RTX 3090 GPU. This protocol avoids counting one-time initialization overhead and makes the baseline and IRA-YOLO comparison reproducible.

**Figure 5:**
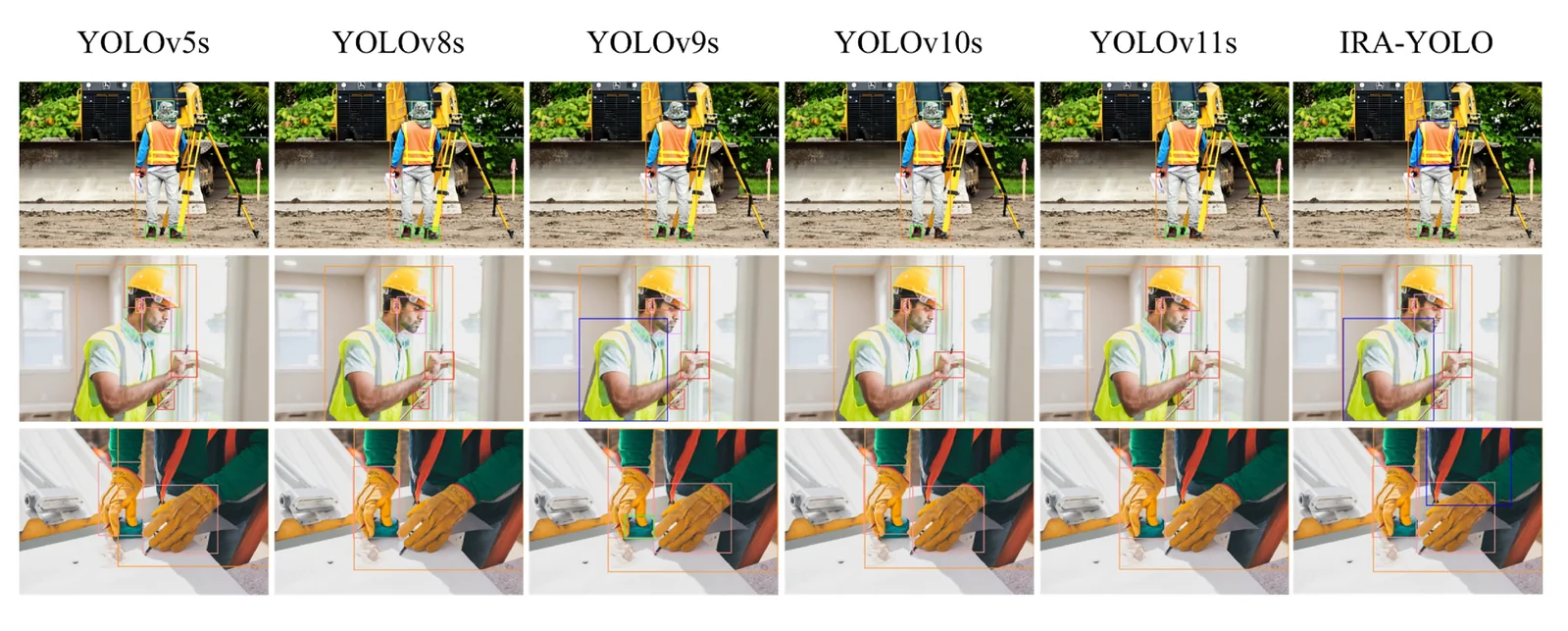
Detection performance comparison of different models on the SH17 validation set. The colored boxes indicate the detected PPE categories and their corresponding predicted locations. The incomplete visual content is caused only by layout cropping for compact presentation and does not affect the scientific understanding of the detection results.

To verify the performance advantage of the proposed IRA-YOLOv11 model, comparative experiments were conducted on the SH17 dataset under identical experimental settings (Figure 5). In addition to the YOLOv11s baseline, the comparison included mainstream YOLO detectors (YOLOv5s, YOLOv8s, YOLOv9s, and YOLOv10s), representative YOLO variants (YOLOX-s and MambaYOLO-T), and the Transformer-based industrial object detector RT-DETR. During validation, the IoU threshold of non-maximum suppression (NMS) was set to 0.7 for all applicable models. The results are reported in Table 5, where the best value for each metric is highlighted in **bold** and the second-best value is underlined.

The experimental results demonstrate that IRA-YOLOv11 achieves comprehensive improvements over the YOLOv11s baseline without substantially increasing computational cost. Its precision reaches 0.757, representing a relative improvement of approximately 3.98% over 0.728, while recall increases from 0.576 to 0.591 (2.60%). The overall mAP@50:95 improves from 0.396 to 0.411 (3.79%). Moreover, mAP@50 and mAP@75 reach 0.633 and 0.446, corresponding to relative gains of 3.77% and 4.69%, respectively. Among all compared methods, IRA-YOLOv11 obtains the best precision, mAP@50:95, and mAP@75, while achieving the second-best recall and mAP@50.

From an efficiency perspective, IRA-YOLOv11 requires 9.83 M parameters and 22.6 GFLOPs. Compared with YOLOv11s, the parameter count increases by only 0.42 M (4.46%) and the computation increases by 1.0 GFLOPs (4.63%), indicating that the accuracy gains are obtained with a modest overhead. Although MambaYOLO-T is the most compact model, IRA-YOLOv11 improves its mAP@50:95 by 0.052. Compared with RT-DETR, IRA-YOLOv11 reduces the parameter count by 69.30% and GFLOPs by 78.16%, while improving precision, mAP@50:95, and mAP@75 by 0.027, 0.019, and 0.040, respectively. These results show that the inter-class relation attention mechanism strengthens category-aware feature extraction and offers a favorable balance between detection accuracy and computational complexity for multi-class PPE detection in industrial scenarios.

From an efficiency perspective, IRA-YOLOv11 requires 9.83 M parameters and 22.57 GFLOPs, while the YOLOv11s baseline requires 9.41 M parameters and 21.58 GFLOPs. Thus, the IRAM adds 0.42 M parameters and 0.99 GFLOPs, corresponding to increases of 4.46% and 4.59%, respectively. The measured single-image latency increases from 5.73 ms to 7.15 ms, and FPS decreases from 174.68 to 139.83. Although this indicates a measurable runtime cost, IRA-YOLOv11 still operates above 100 FPS under the tested RTX 3090 FP32 setting, supporting its real-time applicability for PPE detection.

In Table 5, Params is the number of trainable model parameters and GFLOPs is the number of billions of floating-point operations for one 640 × 640 forward pass. Precision (P=TP/(TP+FP)) measures the proportion of predicted detections that are correct, whereas recall (R=TP/(TP+FN)) measures the proportion of ground-truth instances recovered; mAP@50 is mean average precision at an intersection-over-union threshold of 0.50, mAP@75 uses 0.75, and mAP@50:95 averages mAP over thresholds from 0.50 to 0.95 in increments of 0.05.

MambaYOLO-T provides the lowest parameter count and GFLOPs in this comparison, but its mAP@50:95 is 0.359, compared with 0.411 for IRA-YOLOv11. We did not replace the YOLOv11 backbone with Mamba because this study isolates the effect of inter-class relation attention under a fixed detector backbone. Simultaneously changing the backbone and attention mechanism would confound attribution of the observed gain. Combining the IRAM with a state-space backbone is therefore reserved for a controlled follow-up study.

### 4.3. Ablation Studies

#### 4.3.1. Module Architecture Analysis

We further conducted ablation experiments to investigate the key design choices in the IRAM, as summarized in Table 6. Replacing stacked convolutions with dilated convolutions yields an improvement over the baseline, yet it still underperforms the full design. A possible explanation is that dilated convolutions compromise local spatial continuity, which may be essential for learning coherent class attention maps. Deferring the dimensional transformation to the last layer not only increases computational cost but also weakens the integration of relational information, resulting in lower accuracy. Direct fusion via broadcasting leads to a performance drop of 0.8% relative to the baseline, suggesting that high-level relational semantics and low-level visual embeddings are poorly aligned under naive broadcasting.

#### 4.3.2. Comparison with a GCN-Style Relation Baseline

To directly evaluate whether the improvement comes from relation modeling in general or from the proposed asymmetric dense relation design, we added a GCN-style relation baseline. As shown in Table 7, the baseline keeps the YOLOv11s backbone, neck, training split, image size, optimizer, learning-rate schedule, and detection head unchanged. It replaces the IRAM with a GCNRelation module at the same P3, P4, and P5 feature scales. Specifically, class attention maps are treated as graph nodes, a learnable class-to-class adjacency matrix propagates information among the 17 PPE categories, and the graph-refined class maps are projected back to the original feature dimension through residual fusion.

The GCNRelation baseline reaches 0.408 mAP@50:95, which is 0.012 above YOLOv11s and only 0.003 below IRA-YOLO. This indicates that relation modeling is the primary source of improvement, while the asymmetric dense design of the IRAM yields a comparable accuracy at a slightly higher computational cost.

#### 4.3.3. Background Category Analysis

We further investigated whether incorporating the background class into relation modeling benefits performance, as reported in Table 8. Including the background class degrades performance across all evaluation metrics. A likely explanation is the semantic and visual heterogeneity of background regions, which lack stable relational patterns with foreground PPE categories. Consequently, background attention maps may introduce noise during training and interfere with foreground relation learning. This finding supports restricting relation modeling to foreground categories.

#### 4.3.4. Comparison with Other Attention Mechanisms

To validate the effectiveness of the IRAM, we compared it with several representative attention mechanisms: SEBlock [7], CBAM [8], ECA [20], CoordAttention [21], BAM [22], SimAM [23], and SA-Net [24]. All experiments were conducted under identical settings, with only the attention module replaced to ensure a fair comparison.

Table 9 reports the results. Conventional attention mechanisms (SEBlock [7], CBAM [8], BAM [22], SimAM [23], SA-Net [24]) yield marginal or even negative gains over the baseline. Their core operation, reweighting visual saliency cues such as texture, edges, and channels, proves insufficient for complex industrial scenes, where PPE targets are frequently occluded or visually indistinct. Such methods tend to amplify background noise or over-focus on local patterns while lacking the capacity for cross-category logical reasoning. CoordAttention [21] embeds positional information and slightly improves mAP@50:95 to 0.400, confirming the benefit of spatial awareness. However, it still fails to model co-occurrence constraints (e.g., helmet and head), which limits its gain.

In contrast, the IRAM achieves gains of 1.5% in mAP@50:95 (0.396 → 0.411), 2.3% in mAP50, and 2.0% in mAP75, outperforming all counterparts. The improvement stems from explicit cross-category relation modeling: leveraging logical co-occurrences (e.g., glove–hand, helmet–head) to assist hard-to-detect targets and suppress false detections that violate co-occurrence patterns. This fills the semantic gap of traditional attention mechanisms and suits the strongly correlated multi-class PPE detection task.

**Figure 6:**
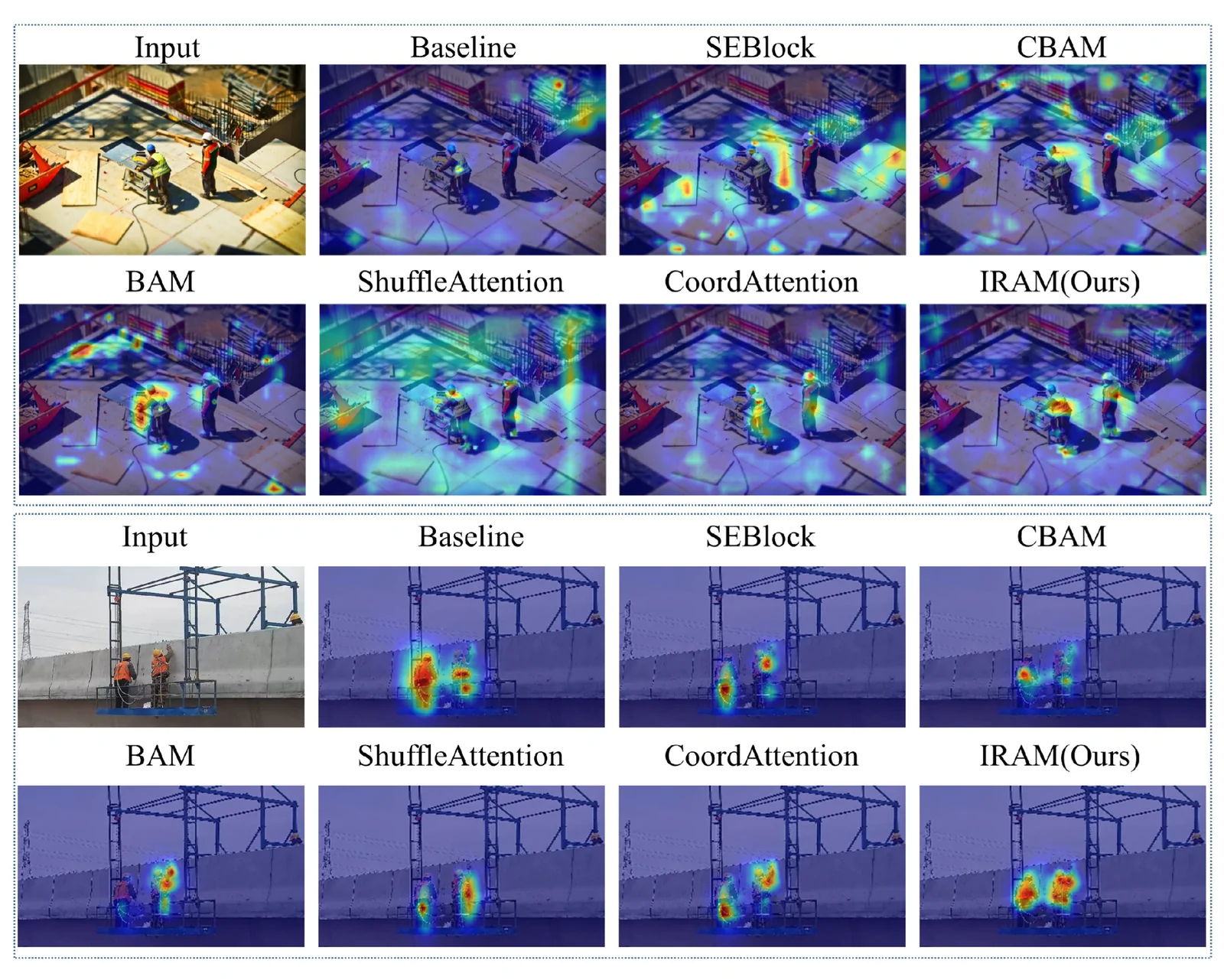
Attention heatmap comparison on an online image and a real-world construction scene. The proposed IRAM demonstrates superior precision and semantic consistency in activating PPE targets. In the heatmaps, warmer colors indicate higher attention responses, while cooler colors indicate lower attention responses.

We further visualize attention maps using GradCAM in Figure 6, which presents comparisons on two representative images: an online image in the top row and a real-world highway construction scene in the bottom row. The baseline suffers from scattered attention and heavy background noise. Channel and spatial attentions (SEBlock, CBAM, BAM, SA-Net) still produce coarse activations with localization errors. CoordAttention focuses on the worker’s body but misses fine-grained PPE such as shoes. In both scenarios, the IRAM precisely and consistently activates helmets, protective clothing, and shoes, demonstrating robust and fine-grained feature focusing, which effectively reduces false detections in complex backgrounds.

### 4.4. Cross-Dataset Generalization

To assess whether the proposed inter-class relation modeling generalizes beyond SH17, we further evaluated IRA-YOLO and the YOLOv11s baseline on CHVG (see Table 3), following the same training process described in Section 4. Both models were initialized with YOLOv11s pretrained weights and evaluated on the same CHVG validation split containing 254 images and 1647 instances. Table 10 reports the results.

On the CHVG validation set, IRA-YOLO attains a precision of 0.874 and a recall of 0.880, exceeding the YOLOv11s baseline by 0.006 and 0.003, respectively. These results indicate that inter-class relation modeling slightly reduces false positives and missed detections under the changed scene distribution. The improvement in classification-oriented metrics further supports this observation: mAP@50 increases from 0.915 to 0.917, and mAP@75 rises more notably from 0.693 to 0.715. The larger gain at mAP@75 suggests that the improved classification confidence also helps produce better-aligned bounding boxes under high IoU requirements.

### 4.5. Per-Class and Fine-Grained Analysis

**Figure 7:**
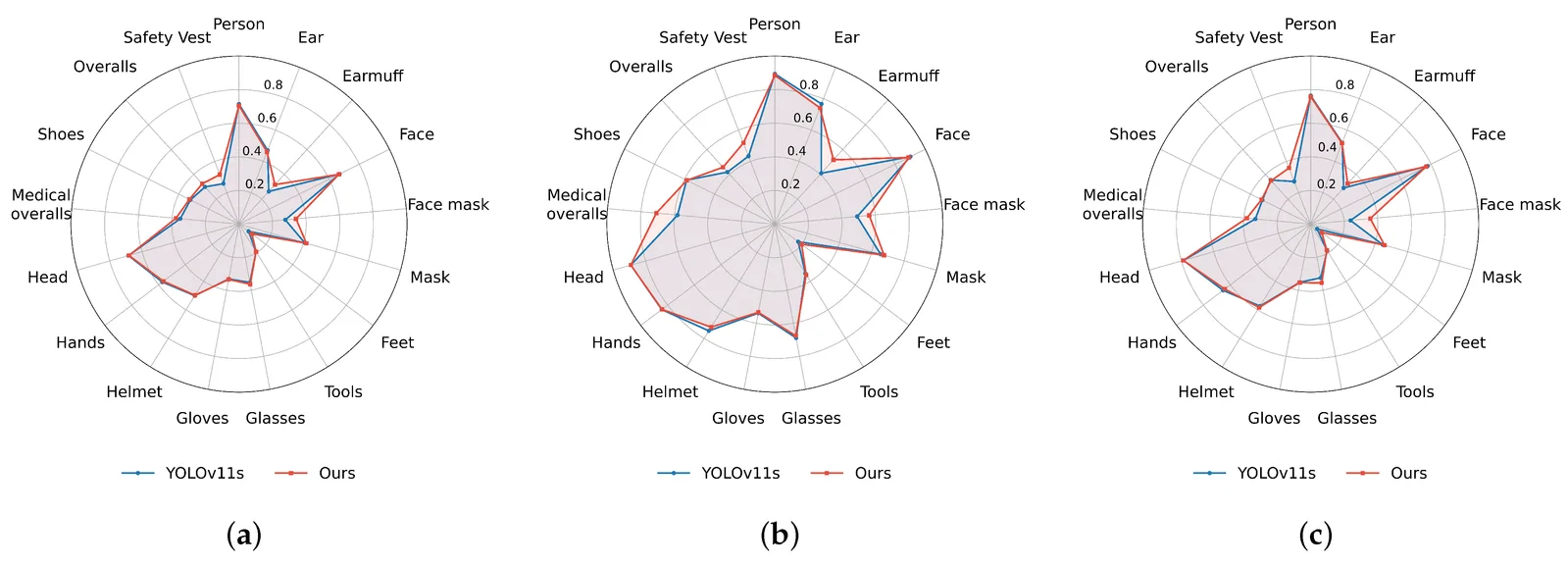
Comparison of detection performance by class on the SH17 validation set: (**a**) mAP@50:95; (**b**) mAP@50; (**c**) mAP@75.

To better understand where the improvements originate, we further compared per-class detection performance between IRA-YOLO and the YOLOv11s baseline on all 17 SH17 categories using radar charts, as shown in Figure 7, to test whether the IRAM specifically improves detection under occlusion, as hypothesized in Section 3.

Figure 7 compares IRA-YOLO against the YOLOv11s baseline across the 17 SH17 categories. Across the mAP@50:95, mAP@50, and mAP@75 radar charts, IRA-YOLO outperforms the baseline on most PPE categories, with particularly pronounced gains on long-tailed categories such as face mask, ear muffs, and safety vest, which intuitively demonstrates the effectiveness of inter-class relation attention for PPE detection. Moreover, the radar profile of IRA-YOLO is noticeably fuller and more balanced than that of the baseline, indicating that the IRAM not only improves overall detection accuracy but also enhances category-wise balance and robustness. This allows the model to more reliably identify PPE instances across categories with highly imbalanced sample sizes, better satisfying the high reliability requirements of complex industrial deployment scenarios.

### 4.6. Training Curves and Taxonomy Scaling

**Figure 8:**
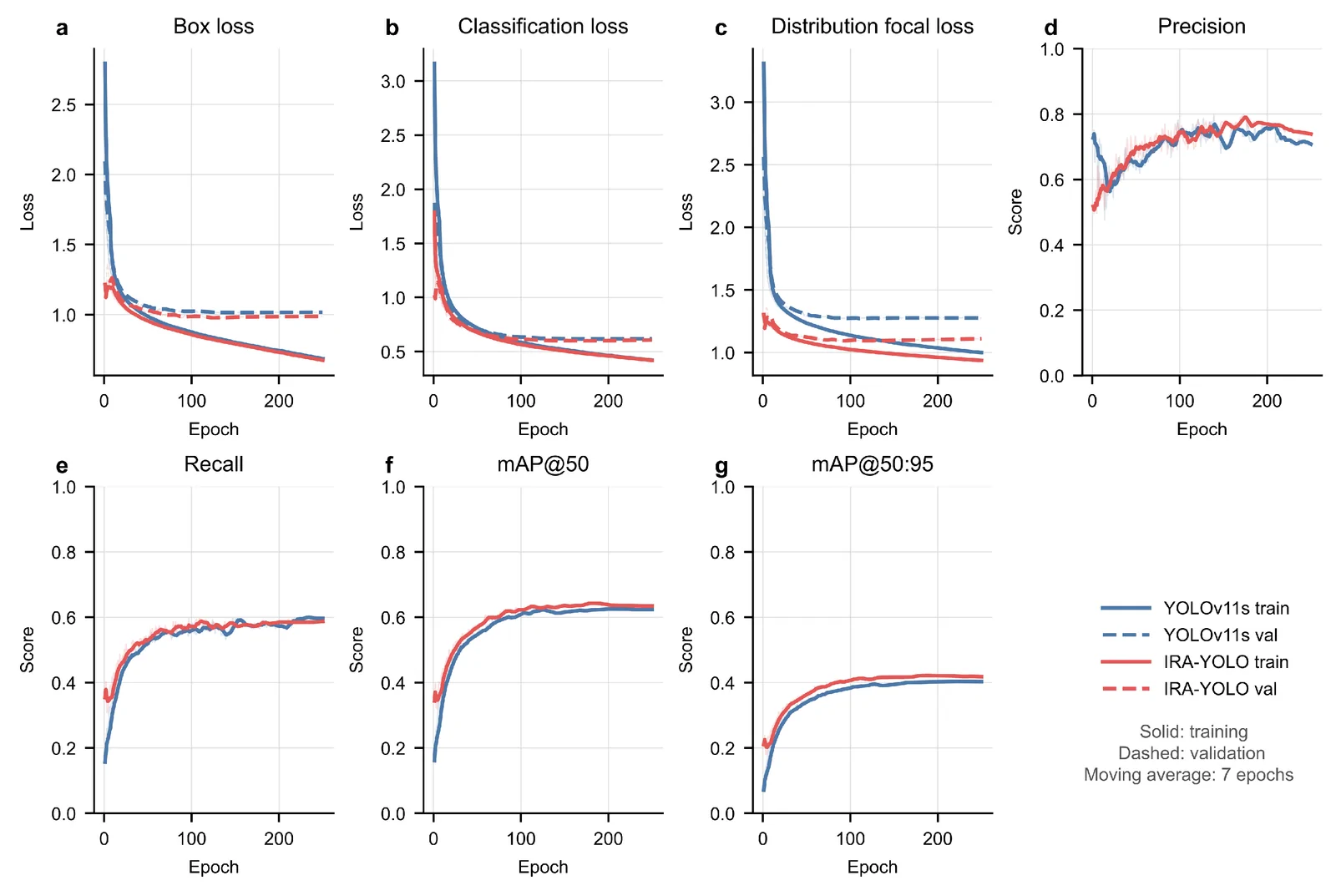
Training and validation curves of YOLOv11s and IRA-YOLO. Panels (**a**–**c**) show box-regression, classification, and distribution-focal losses, respectively. Panels (**d**–**g**) show validation precision, recall, mAP@50, and mAP@50:95. Solid lines denote training losses and dashed lines denote validation losses for the loss panels; the metric panels show validation metrics.

To make the optimization process more transparent, we exported the epoch-wise learning curves of YOLOv11s and IRA-YOLO, as shown in Figure 8. Because object detection has no single classification-accuracy field, the accuracy-related curves report validation precision, recall, mAP@50, and mAP@50:95, while the loss curves report training and validation box-regression, classification, and distribution-focal losses. The two curves show broadly comparable convergence behavior, indicating that the additional inter-class relation modeling does not destabilize training. We therefore use the aggregate validation metrics in Table 5 as the primary performance comparison and use the training curves as diagnostic evidence for optimization traceability.

We also define condition-stratified subsets from the fixed SH17 validation split, as shown in Table 11. The condition-stratified evaluation shows a mixed pattern. IRA-YOLO improves mAP@50:95 on medium-scale and large-scale images by 0.007 and 0.021, respectively, suggesting that inter-class relation modeling is more beneficial when PPE instances retain sufficient spatial evidence. On occluded and cluttered images, the differences are small (−0.004 and −0.002), indicating broadly comparable robustness under these challenging conditions. However, IRA-YOLO underperforms on small-scale and non-occluded subsets, with decreases of 0.014 and 0.049 in mAP@50:95. Therefore, the stratified experiment supports a scale-dependent benefit rather than a universal improvement across all conditions, and we treat occlusion robustness as a design motivation instead of a confirmed condition-specific advantage.

When the taxonomy expands, the ordered pairwise relation representation scales approximately with $N_{c}^{2}$, while the class-specific projection scales with $N_{c}$, as shown in Table 12. We evaluated the isolated three-scale IRAM tensor path at $N_{c}=17$, 50, and 100 using the actual P3/P4/P5 shapes (128,80,80), (256,40,40), and (512,20,20) on an RTX 3090. With batch size one, FP32 precision, 50 warm-up iterations, and 200 synchronized timed runs, mean latency increased from 1.421 ms to 3.815 ms and 13.397 ms. Incremental peak allocated GPU memory increased from 39.79 MB to 311.73 MB and 1225.59 MB, while complexity increased from 0.985 to 2.733 and 9.069 GFLOPs. For the 17-class SH17 setting, the pairwise path is therefore a small fraction of the end-to-end cost. For 50–100-class taxonomies, the quadratic term dominates and sparse or factorized relation representations become necessary; we regard this as a natural extension rather than a limitation of the current SH17-focused design.

## 5. Conclusions

This paper presents IRA-YOLO, a detection framework that introduces explicit inter-class relation modeling for PPE detection in industrial scenes. Motivated by the observation that conventional attention modules seldom capture semantic dependencies among PPE categories, we propose the Inter-Class Relation Attention Module (IRAM), which generates class-specific attention maps via subspace projection, learns pairwise inter-class relations through stacked grouped convolutions, and fuses the relational priors back through a residual connection. Experiments on the SH17 dataset demonstrate that IRA-YOLO outperforms YOLOv11s across all evaluation metrics and surpasses seven representative attention mechanisms. The cross-dataset evaluation on CHVG further shows improvements over YOLOv11s in precision, recall, mAP@50:95, mAP@50, and mAP@75, indicating that the proposed relation modeling remains effective under changes in category taxonomy and scene distribution. The gains stem from the model’s ability to exploit structured co-occurrence patterns among PPE categories—for instance, leveraging the presence of a head to assist helmet detection, which provides semantic guidance beyond appearance-based attention while preserving the efficiency of the YOLO architecture. Future work will explore temporal relation modeling across video frames, low-shot adaptation for newly introduced PPE categories, and evaluation of inference efficiency on embedded or edge-computing hardware to further support industrial deployment.

## Figures and Tables

**Table 1:** Qualitative comparison of relation/attention modeling strategies. IRAM is proposal-free and models asymmetric inter-class relations directly at the feature-map level.

Method	Proposal-Free	Inter-Class Relation	Asymmetric	Complexity
SENet [7]/CBAM [8]	✓	×	–	Low
GCN-style relation baseline [9]	✓	✓	×	Medium
Relation Network [10]	×	✓	✓	Medium–High
Transformer self-attention [28]	✓	✓	✓	High (quadratic)
IRAM (Ours)	✓	✓	✓	Low

*Note:* ✓ indicates that the corresponding strategy is used or supported; × indicates that the strategy is not used or not supported; – indicates that the item is not applicable.

**Table 2:** Notation and tensor dimensions used in the IRAM formulation.

Symbol	Dimension
*F*, $F_{\mathrm{out}}$	$R^{C_{\mathrm{in}}\times H\times W}$
$F^{\prime }$	$R^{d\cdot N_{c}\times H\times W}$
$X_{i}$	$R^{d\times H\times W}$
$A_{i}$, $A_{i}^{\prime }$	$R^{1\times H\times W}$
*A*, $A^{\prime }$	$R^{N_{c}\times H\times W}$
$R_{i,j}$	$R^{1\times H\times W}$
$R_{i}$	$R^{N_{c}\times H\times W}$
R	$R^{N_{c}\cdot N_{c}\times H\times W}$
$A_{\mathrm{proj}}^{\prime }$	$R^{C_{\mathrm{in}}\times H\times W}$

**Table 3:** Comparison of representative PPE detection datasets. SH17 provides the largest category coverage and the most diverse annotation scope among the compared benchmarks.

Dataset	PPE Categories	Number of Categories	Number of Images
SWHD [12]	Helmet	2	7581
SHD [2]	Helmet	3	5000
SHW [12]	Helmet	1	7581
CHV [12]	Helmet, Vest	6	1330
CHVG [12]	Helmet, Vest, Glasses	8	1699
Pictor-v3 [12]	Hat, Vest	3	784
SH17 [12]	Multiple	17	8099

**Table 4:** Real-time inference efficiency of YOLOv11s and IRA-YOLO under the same evaluation protocol.

Model	Params (M)	GFLOPs	Latency (ms)	FPS
YOLOv11s	9.41	21.58	5.73	174.68
IRA-YOLO	9.83	22.57	7.15	139.83
Δ	+0.42	+0.99	+1.42	−34.85

**Table 5:** Comparison of experimental results with other models on the SH17 dataset.

Model	Params	GFLOPs	P	R	mAP@50:95	mAP@50	mAP@75
YOLOv11s [11]	9.41 M	21.6	0.728	0.576	0.396	0.610	0.426
YOLOv5s [3]	7.05 M	16.1	0.756	0.531	0.350	0.586	0.368
YOLOv8s [4]	9.83 M	23.6	0.744	0.554	0.399	0.609	0.434
YOLOv9s [5]	6.20 M	22.8	0.752	0.579	0.408	0.624	0.436
YOLOv10s [6]	8.04 M	24.8	0.706	0.571	0.396	0.602	0.430
YOLOX-s [17]	8.94 M	26.8	0.738	0.498	0.338	0.570	0.353
MambaYOLO-T [18]	**5.98 M**	**13.6**	0.726	0.531	0.359	0.569	0.386
RT-DETR [19]	32.02 M	103.5	0.730	**0.622**	0.392	**0.635**	0.406
Ours (IRA-YOLOv11)	9.83 M	22.6	**0.757**	0.591	**0.411**	0.633	**0.446**

**Table 6:** Ablation study of the IRAM design. The full configuration yields the best overall detection accuracy among the tested variants. The “Dilated Conv (r=1,2,3)” row denotes one sequential three-layer variant whose dilation rates are 1, 2, and 3, respectively.

Configuration	mAP@50:95	mAP50	mAP75
Baseline	0.396	0.610	0.426
+Dilated Conv (*r* = 1, 2, 3)	0.400	0.619	0.433
+Dim Transform	0.407	0.628	0.437
+Broadcasting Enhancement	0.388	0.600	0.416
Full IRAM (Ours)	**0.411**	**0.633**	**0.446**

*Note:* Bold values indicate the best performance for each metric.

**Table 7:** Comparison between YOLOv11s, the GCN-style relation baseline, and IRA-YOLO on SH17. The GCN-style baseline uses the same insertion positions and 400-epoch training protocol.

Model	Relation Module	Params	GFLOPs	mAP@50:95	FPS
YOLOv11s	None	9.41 M	21.58	0.396	174.68
YOLOv11s + GCNRelation	GCN-style class graph	9.82 M	22.34	0.408	143.58
IRA-YOLOv11	Asymmetric dense IRAM	9.83 M	22.57	0.411	139.83

**Table 8:** Effect of including the background class in relation modeling. Restricting relation modeling to foreground categories yields better performance across all metrics.

Category Scope	Number of Categories	mAP@50:95	mAP50	mAP75
Foreground Only	$N_{c}$	0.411	0.633	0.446
+Background	$N_{c}+1$	0.399	0.615	0.432

**Table 9:** Comparison with representative attention mechanisms on the SH17 dataset. All methods were trained under identical settings, and the best result in each column is highlighted in **bold**. Higher mAP indicates better detection performance.

Method	mAP@50:95	mAP50	mAP75
YOLOv11s (Baseline)	0.396	0.610	0.426
+SEBlock [7]	0.390	0.605	0.423
+CBAM [8]	0.392	0.612	0.413
+ECA [20]	0.395	0.614	0.432
+BAM [22]	0.391	0.609	0.422
+SimAM [23]	0.392	0.609	0.423
+SA-Net [24]	0.392	0.609	0.418
+CoordAttention [21]	0.400	0.618	0.439
**+ IRAM (Ours)**	**0.411**	**0.633**	**0.446**

**Table 10:** Cross-dataset generalization results on the CHVG validation set.

Model	Precision	Recall	mAP@50:95	mAP@50	mAP@75
YOLOv11s (Baseline)	0.868	0.877	0.599	0.915	0.693
IRA-YOLO (Ours)	**0.874**	**0.880**	**0.617**	**0.917**	**0.715**

*Note:* Bold values indicate the best performance for each metric.

**Table 11:** Condition-stratified SH17 comparison.

Subset	Images	YOLOv11s mAP@50:95	IRA-YOLO mAP@50:95	Δ
Occluded	1541	0.420	0.416	−0.004
Non-occluded	79	0.184	0.136	−0.049
Small scale	578	0.336	0.322	−0.014
Medium scale	901	0.516	0.522	+0.007
Large scale	141	0.564	0.586	+0.021
Cluttered	1112	0.420	0.418	−0.002
Non-cluttered	508	0.392	0.382	−0.010

**Table 12:** Scaling of the isolated three-scale IRAM tensor path with the number of categories.

$N_{c}$	*d* at P3/P4/P5	Params (M)	GFLOPs	Mean ± SD (ms)	P95 (ms)	Peak MB
17	8/16/32	0.4228	0.9853	1.421 ± 0.047	1.478	39.79
50	2/4/8	0.6424	2.7326	3.815 ± 0.118	3.850	311.73
100	1/2/4	1.6579	9.0693	13.397 ± 0.327	13.526	1225.59

## Data Availability

The data used in this study are publicly available from the SH17 dataset.
